# Do acute inflammatory cytokines affect 3- and 12-month postoperative functional outcomes–a prospective cohort study of 12 patients with proximal tibia fractures

**DOI:** 10.1186/s12891-021-04207-7

**Published:** 2021-04-10

**Authors:** Imran Jamal Iversen, That Minh Pham, Hagen Schmal

**Affiliations:** 1 Department of Orthopaedic Surgery and Traumatology, Odense University Hospital, Odense, J.B. Winsløws Vej 4, 5000 Odense, Denmark; 2 University of Southern Denmark, Odense, Denmark; 3 Department of Clinical Research, University of Southern Denmark, Odense, Denmark; 4 Clinic of Orthopedic Surgery, Medical Center—University of Freiburg, Faculty of Medicine, University of Freiburg, Freiburg, Germany; 5 OPEN, Odense Patient data Explorative Network, Odense University Hospital/Institute of Clinical Research, University of Southern Denmark, Odense, Denmark

**Keywords:** Fracture, Osteoarthritis, Post-traumatic, Inflammatory, Intra-articular, Synovial fluid, Clinical outcome

## Abstract

**Background:**

Patients with intra-articular fractures tend to develop post-traumatic osteoarthritis (PTOA). The initial inflammatory response with elevation of inflammatory cytokines following joint trauma might be responsible for triggering cartilage catabolism and degradation. We aimed to identify and quantify cytokine levels in fractured and healthy knee joints and the correlation of these cytokines with clinical outcomes.

**Methods:**

In this prospective cohort study, synovial fluid and plasma were collected from 12 patients with proximal intra-articular tibia fractures before surgery. The concentration of sixteen inflammatory cytokines, two cartilage degradation products and four metabolic mediators where measured, comparing the acute injured knee with the healthy contralateral knee. Patients were evaluated 3- and 12-months after surgery with clinical parameters and radiographical scanning. Non-parametrical Wilcoxon rank-sum and Spearman tests were used for statistical analysis, and a *P*-value below 0.05 was considered significant.

**Results:**

We found an elevation of the pro-inflammatory cytokines IL-1β, IL-2, IL-6, IL-8, IL-12p70, TNF-α, IFN-y, MMP-1, MMP-3, and MMP-9 and a simultaneous elevation of the anti-inflammatory cytokines IL-1RA, IL-4, IL-10, and IL-13 in the injured knee. Several pro- and anti-inflammatory cytokines and metabolic mediators were correlated with clinical outcomes 12 months after surgery, especially with pain perception.

**Conclusions:**

Our results support that an inflammatory process occurs after intra-articular knee fractures, which is characterized by the elevation of both pro- and anti-inflammatory cytokines. There was no sign of cartilage damage within the timeframe from injury to operation. We found a correlation between the initial inflammatory reaction with clinical outcomes 12 months after surgery.

**Supplementary Information:**

The online version contains supplementary material available at 10.1186/s12891-021-04207-7.

## Background

Osteoarthritis (OA) is one of the reasons for chronic pain and disability worldwide [1]. The physical impairment caused by OA of a single lower extremity joint can disrupt the quality of life similar to the disruption caused by end-stage kidney disease or heart failure [2]. Up to 40% of patients with joint-affecting trauma develop OA within 10 years, and OA has been associated with previous biomechanical treatment [1, 3].

Therefore, the current fracture treatment principle is anatomical reconstruction of the joint surface combined with functional and adequate aftercare. Fracture complexity, high body mass index, age over 30 years, and synovial cytokine regulation have been assumed to significantly influence the outcome [3, 4].

Previous studies indicate that the initial inflammatory response following joint trauma might be responsible for triggering cartilage catabolism and cartilage degradation [3, 5]. Unfortunately, due to the lack of blood supply, cartilage regenerates much less efficiently than bone. Nonetheless, the exact inflammatory mechanism(s) and level of cytokine involvement that lead to the progression from initial injury to post-traumatic OA (PTOA) requires further investigation before we can supplement current surgical practice to improve outcomes following intra-articular fractures [2, 6, 7].

The purpose of this project is to investigate the presence of differently regulated biomarkers in the joint space in the injured and healthy knees of patients with an intra-articular proximal tibia fracture. The project also investigated if the identified biomarkers found in intra-articular proximal tibia fractures correlate with short- and middle-term clinical symptoms after surgery.

In addition, as explorative outcomes, we investigated if cytokine levels in intra-articular proximal tibia fractures correlate with cytokine levels in plasma and if the fracture classification and fracture reduction correlate with the clinical outcomes.

## Methods

In this prospective cohort study, patient recruitment was carried out in the Department of Orthopedic Surgery at Odense University Hospital, while biomarker analyses took place in the Department of Neurobiology Research.

All patients diagnosed with a proximal intra-articular tibia fracture hospitalized in Odense University Hospital between October 2017 and March 2019, were recruited for this study.

**Fig. 1:**
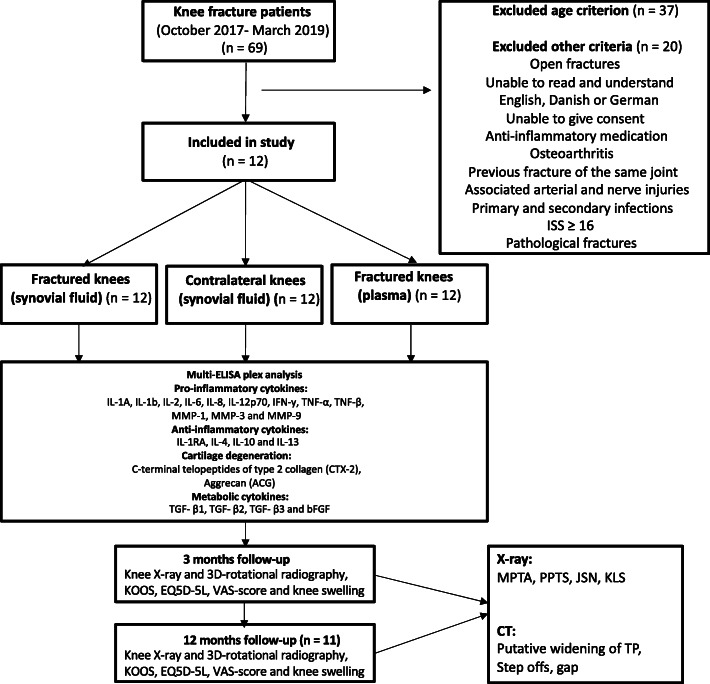
Flowchart of method

The inclusion criteria were the existence of a fracture involving the knee (location AO 41 type B or C) requiring open or closed reduction and internal or external fixation, age between 18 and 60 years, operation within 2 weeks after the fracture occurred, being able to read and understand English, German or Danish, and able to give informed consent. The exclusion criteria were open fractures Gustilo Anderson classification grade II or III, compartment syndrome, previous fracture of the same joint, signs of osteoarthritis verified on X-ray (Kellgren Lawrence Score ≥ 3), multiple injury patients with an Injury Severity Score (ISS) ≥ 16, associated arterial and nerve injuries, primary or secondary infections or osteomyelitis, pathological fractures, and receiving anti-inflammatory medications (Fig.1).

After the operation, patients were mobilized with 20 kg partial weight bearing for 6 weeks. Afterwards, weight bearing was increased depending on X-ray and pain perception. After 3 months, all patients could tolerate full weight bearing, at least for small distances.

### Collection of synovial fluids and blood samples from knee fractured patients

Prior to surgery, synovial fluid (SF) was collected from the fractured knee (*n* = 12) and the same patient’s contralateral knee. The patient was positioned in a supine position, and the disinfection of both knees followed our department’s guidelines. A 1.5 × 50 mm needle was inserted in the joint line using the lateral approach. Once the needle was in the joint space, a volume of 5.0 ml isotonic saline was injected into the joint place and mixed before retraction, optimizing the sufficient volume needed. This procedure was mainly performed by two surgeons (TMP/HS), and in the case that other surgeons collected the samples, an x-ray was used for assistance. SF was collected, transferred in a 10 ml glass, and transported to the Department of Clinical Biochemistry and Pharmacology for centrifugation at 2000 rpm (RPM) for 15 min, and storage at minus 80 degrees Celsius within 2 h. Blood samples were also collected in ethylenediaminetetraacetic acid (EDTA) collection tubes for comparison (Fig.1) and underwent same centrifugation and storage as the SF.

### Cytokine analysis

To discriminate between the fracture and the contralateral healthy joint, a proteomics analysis was used to identify the classical pro-inflammatory cytokines and the cytokines being involved in the extracellular breakdown and cartilage degeneration. A custom multi-electrochemiluminescence immunoassay (ELISA) Plex including, IL-1 alfa (α), IL-1 receptor antagonist (RA), IL-1 beta (β), IL-2, IL-4, IL-6, IL-8, IL-10, IL-12p70, IL-13, interferon gamma (IFN-γ), tumor necrosis factor alfa (TNF-α), and TNF-β was used (Mesoscale, Rockville, MD). Matrix metalloproteinase (MMP)-1, MMP-3, and MMP-9 were measured using a human MMP 3-Plex Ultra-sensitive Kit (Mesoscale, Rockville, MD). Basic fibroblast growth factor (b-FGF) and transforming growth factor (TGF)-β1, TGF-β2, and TGF-β3 were measured by V-Plex b-FGF kit and U-PLEX TGF-β Combo kit (Mesoscale, Rockville, MD). Aggrecan (ACG) and c-terminal telopeptides of type 2 collagen (CTX-2) SF levels were analyzed by ELISA (Fig.1) (MyBiosource, VersaMax™). All samples were run in duplex and done according to the manufacturer’s instruction.

### Parameters

The following epidemiological parameters were collected: age, sex, body mass index (BMI), the American Society of Anesthesiologists (ASA) physical classification system, the time between the fracture occurred and operation, internal or external fixation, the Schatzker classification of tibial plateau fractures [8] and the classification of injury according to Arbeitsgemeinschaft für Osteosynthesefragen (AO) standards (41B and 41C).

All patients were evaluated at 3 and 12 months after surgery according to the following clinical parameters: pain (visual analog scale), swelling (measurement of the circumference of the knee in cm), knee X-ray, 3D-rotational tomography, and validated scores (Knee injury and Osteoarthritis Outcome Score (KOOS) and the Euroqol 5D questionnaire (EQ. 5D)) [9, 10].

### Radiographic analysis of X-ray

PTOA is evaluated using the Kellgren-Lawrence scale (KLS), assessed after one year [11]. This method has recently been described and validated in a retrospective cohort [12].

**Fig. 2:**
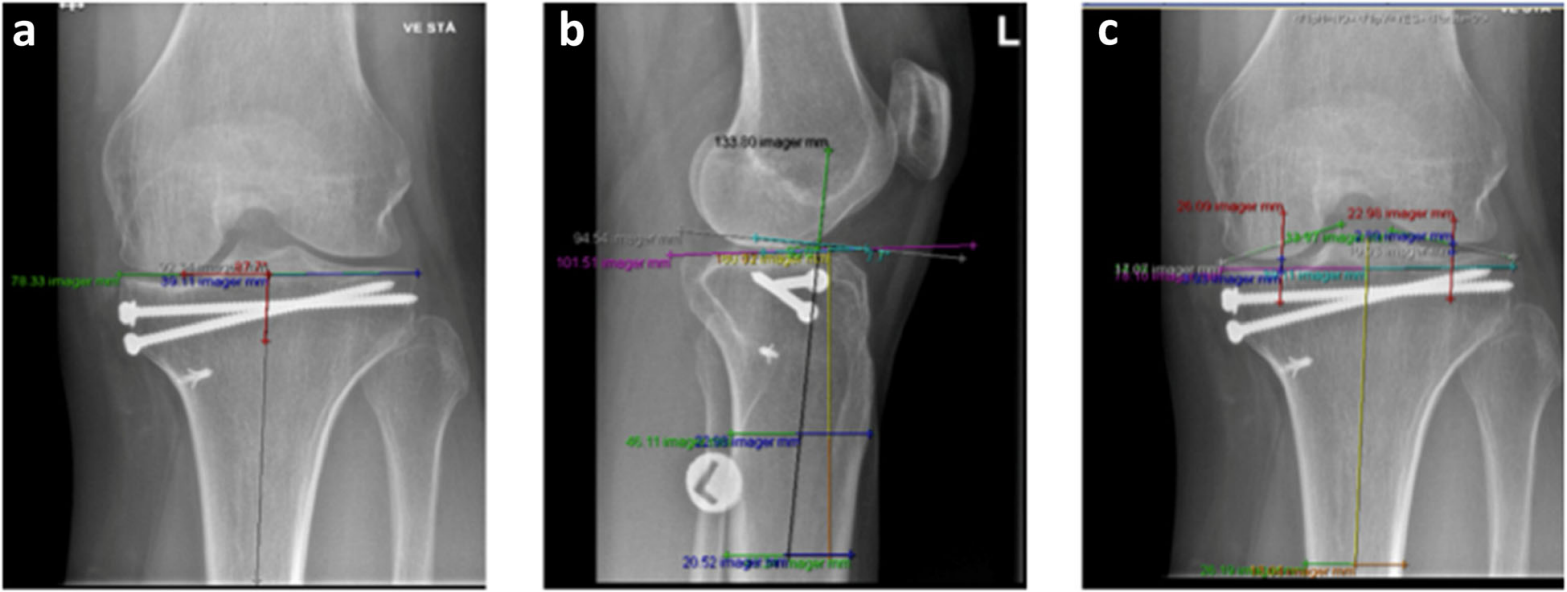
Angle and joint space criteria measured on postoperative weight bearing X-ray 12 months after knee surgery. **2a**: MPTA: This is measured by drawing a line in parallel with the tibial joint surface and measuring the medial angle between this line and a line drawn along the long axis of the tibia. Values > 5° from 90° were considered to be malalignment [12, 13]. **2b**: PPTS angle: Longitudinal axis of the tibia (LAT, as black line) equals the line passing through two points located in the center of the anterior-posterior width of the tibia at 6 and 10 cm apart on the proximal diaphysis (the two distal green lines). The angle formed between the line perpendicular to the LAT (grey) and the line passing through the highest anterior and posterior points of the tibial plateau (purple) represents the PPTS angle. The slope was defined as positive if the line passing through the highest posterior point of the tibial plateau was below the perpendicular line [14]. **2c**: Medial and lateral JSN: Line between midpoints is the long axis of tibial shaft (LATS) (yellow line). Midpoints (white line) of the medial and lateral compartments were identified by using the line between the medial and lateral edges of each compartment of the tibial plateau (green line). Midpoint lines (red lines) were drawn parallel to the LATS through the midpoint of each compartment. The distance between the femoral and tibial intersections was measured (blue line). This is the JSN measurement of each compartment respectively [15]

Varus and valgus malalignments are measured by the medial proximal tibial angle (MPTA) on anteroposterior radiographs [12, 13]. Proximal posterior tibial slope (PPTS) was measured on lateral radiographs [14]. Joint space narrowing (JSN) is measured by identifying the midpoint of the tibial shaft and the proximal intercondylar midpoint on anteroposterior radiographs [15]. Measurements were performed on radiographs taken after 3 and 12 months. The difference between 3- and 12-month data were used for the correlation analysis (Figs.1 and 2).

### Radiographic analysis of 3D-rotational CT-scanning

**Fig. 3:**
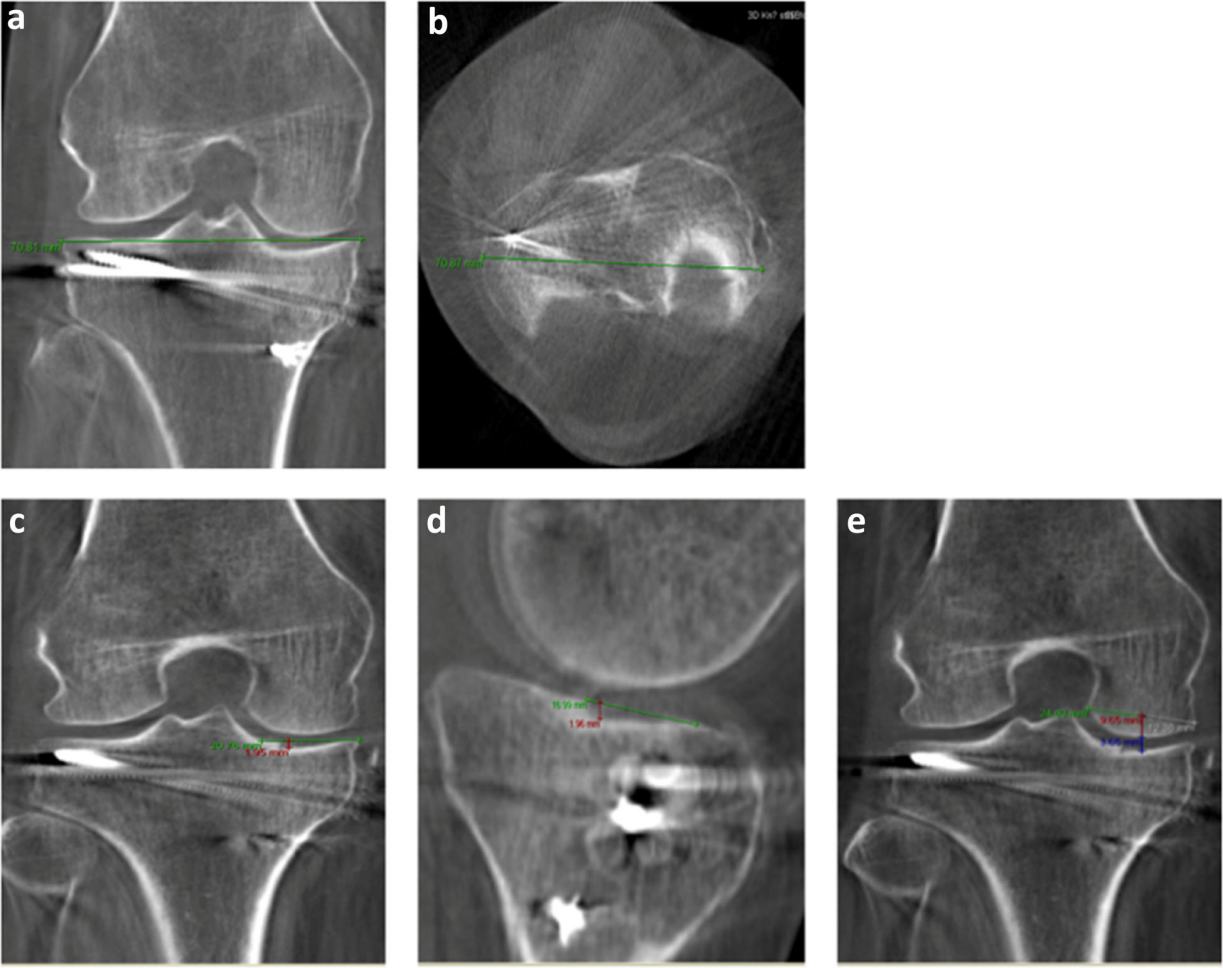
Plateau, step-off and gap criteria measured on postoperative CT 12 months after knee surgery. **3a and 3b**, Widening of tibial plateau. **3c and 3d**, Step offs: Drawing a line from the lateral edge of the affected compartment of the tibial plateau into the intact tibial plateau line (ITPL, green line) and subsequently drawing a line perpendicular from the ITPL down to the deepest point of the step-off (red line). Mean distance between planes is the distance of the step-off. **3e**, Gap: Drawing a line between the lateral and medial edge of the femur condyle (green line). Midpoint is then located (white line). Perpendicular line (red line) is drawn downwards through femur and tibia. The distance between the femur and tibia is the gap (blue line)

The putative widening of the tibial plateau in the coronal and axial bone window is measured by making two marks at the tibial plateau at its widest. The distance between these marks is equal to the putative widening. The mean of both measurements is the final length. The remaining step-offs were visualized in the coronal and sagittal plane. The medial/lateral gap was measured on the same CT slide as the step-off (Figs.1,3). Measurements were performed on CT-scans performed after 3 and 12 months. The difference between 3- and 12-month data will be used for the correlation analysis.

### Statistical analysis

The quantile-quantile (q-q) plot test of all cytokines showed a non-parametrical pattern. The Wilcoxon rank-sum test was used to detect the differences in cytokine levels between the fractured knee and contralateral healthy knee. The correlation analysis was performed using the Spearman test (spearman correlation coefficient = ρ) for non-parametric data. Results are presented with a *P*-value of < 0.05, which is considered significant, in the form of median with interquartile range (IQR). All statistical analyses were performed using STATA MP 16.

## Results

Twelve patients with proximal intra-articular tibia fractures were included in this prospective cohort. The cohort included 5 men and 7 women (mean age 44.1 years ±11.5 years) with a mean BMI of 27.8 ± 6.8. The time from injury to operation when the SF was collected had a mean of 5.8 days, extending from 1 to 13 days. During the 12 months of follow up, 5 patients were re-operated or underwent other operations (Supplement 5). Fracture classifications are presented in Table 1 with the baseline characteristics, and the mean scores and additional clinical outcomes 3 and 12 months after surgery are presented in Table 2. All values are presented as raw *P*-values without multiple comparison correction and should be interpreted with caution.

**Table 1:** Baseline characteristics

Sex: n (%)	
Male	5 (41.6%)
Female	7 (58.3%)
Age: mean (SD)	44.1 (11.5)
BMI: mean (SD)	27.8 (6.8)
ASA score: n (%)	
1	4 (33.3%)
2	7 (58.3%)
3	1 (8.3%)
4	0 (0%)
Schatzker classification: n (%)	
1	1 (8.3%)
2	5 (41.6%)
3	0 (0%)
4	1 (8.3%)
5	1 (8.3%)
6	4 (33.3%)
AO classification: n (%)	
41B	7 (58.3%)
41C	5 (41.6%)
Mean time from injury to operation (min.-max.)	5.8 days (1–13 days)
Post-operative complications: n (%)	2 (16.6%)
Method of operation: n (%)	
Internal fixation	8 (66.6%)
External fixation	4 (33.3%)
Mobilization before injury: n (%)	
With help	0 (0%)
No help	12 (100%)
Able to walk more than 500 m before injury: n (%)	
Yes	12 (100%)
No	0 (0%)

Abbreviation

AO: Arbeitsgemeinschaft für Osteosynthesefragen SD: standard deviation BMI: body mass index ASA:

American Society of Anesthesiologists Classification

**Table 2:** Mean scores and additional clinical outcomes 3 and 12 months after knee surgery

KOOS: 3 months (mean ± SD)	61.8 ± 13.5
KOOS: 12 months (mean ± SD)	70.4 ± 17.7
EQ-5D-5L index 12 months (mean ± SD)	0.76 ± 0.14
Kellgren-Lawrence score: 12 months	2 ± 0.7
Joint space reduction difference after 12 months	
Medial (mean ± SD) (mm)	0.36 ± 3.2
Lateral (mean ± SD) (mm)	− 0.04 ± 3.0
EQ 5 D VAS: 3 months (mean ± SD)	70 ± 24.0
EQ 5 D VAS: 12 months (mean ± SD)	79.5 ± 21.2
VAS-score: 3 months at rest (mean ± SD)	1.5 ± 1.7
VAS-score: 3 months in activity (mean ± SD)	3.3 ± 2.4
VAS-score: 12 months at rest (mean ± SD)	0.8 ± 1.6
VAS-score: 12 months in activity (mean ± SD)	2.1 ± 3.0
Swelling: 3 months from surgery (mean ± SD) (cm)	1.3 ± 0.6
Swelling: 12 months from surgery (mean ± SD) (cm)	0.54 ± 0.52
Mobilization after 3 months	
1: Still need assistive devices	33.3%
2: Can walk without assistive devices	11.1%
3: Can walk more than 500 m without assistive devices	55.5%
4: Returned to normal mobilization	0%
Mobilization after 12 months	
1: Still need assistive devices	0.0%
2: Can walk without assistive devices	0.0%
3: Can walk more than 500 m without assistive devices	72.7%
4: Returned to normal mobilization	27.2%

KOOS (range from 0 to 100%), EQ. 5D-5L Danish index (range from − 0.624–1, with 0, 1, and negative values corresponding to death, full health, and health states worse than death, respectively. Kellgren-Lawrence score (grade 0–4)

Abbreviation

KOOS: Knee injury and Osteoarthritis Outcome Score VAS: visual analog scale SD: standard deviation

### Cytokine levels in fractured knee joints were significantly increased compared to healthy contralateral knee joints

We found a statistically significant increase in cytokine levels in the injured knees of patients with acute intra-articular proximal tibia fractures compared to their contralateral healthy knee. Nearly all pro-inflammatory cytokines, including IL-1β, IL-2, IL-6, IL-8, IL-12p70, TNF-α, IFN-y, MMP-1, MMP-3, and MMP-9, were increased. IL-6 and IL-8 had the largest fracture/contralateral knee cytokine ratio. All anti-inflammatory cytokines were increased, including IL-1RA, IL-4, IL-10, and IL-13. Only the metabolic mediator cytokine TGF-β3 had a significant decrease in its level. No significant differences were found between pro-inflammatory cytokines IL-1α and TNF-β, cartilage degradation products ACG and CTX-2 and the metabolic cytokine mediators b-FGF, TGF-β1, and TGF-β2 (Table 3).

**Table 3:** Cytokines levels in fractured knees compared to healthy contralateral knee

	Cytokine levels	Fractured knees (pg/mL) Median (IQR)	Contralateral knees (pg/mL) Median (IQR)	*P*-value (z-value)	Cytokine ratio (fractured/contralateral)	Serum (pg/ml) Median (IQR)
Pro-inflammatory	IL-1α	0.01345 (0.01226)	0.007 (0.000)	0.502 (− 0.67)	1.30	0.00942 (0.02051)
	IL-1β	5.16 (2.86)	0.127 (0.000)	**0.0001 (− 3.84)**	40.61	0.665 (0.465)
	IL-2	3.43 (2.21)	0.47 (0.00)	**0.0003 (− 3.65)**	7.34	0.710 (0.005)
	IL-6	1630.62 (865.54)	0.216 (0.000)	**0.0001 (−3.82)**	2618.14	5.93 (3.17)
	IL-8	379.21 (309.79)	1.62 (0.59)	**0.0002 (−3.67)**	212.85	3.35 (4.41)
	IL-12p70	6.95 (5.16)	0.93 (0.00)	**0.0001 (−3.88)**	7.15	0.71 (0.06)
	TNF-α	8.92 (7.77)	0.403 (0.000)	**0.0001 (−3.84)**	20.66	2.61 (1.42)
	TNF-β	0.0000419 (0.000116)	0.000046 (0.00000)	1.00 (0.00)	2.47	0.0000426 (7.00e-07)
	IFN-y	28.81 (24.51)	3.81 (0.00)	**0.0001 (−3.88)**	7.68	10.65 (12.07)
	MMP-1	426,290.2 (804,764.8)	145.14 (7996.7)	**0.0055 (−2.77)**	74.49	1649.53
	MMP-3	342,739.1 (675,480.8)	79,747.3 (138253)	**0.026 (−2.21)**	5.65	6812.93 (1817.63)
	MMP-9	76,405.31 (56,070.87)	122.50 (4300.56)	**0.047 (−1.98)**	2.86	49,956.95 (79,704.52)
Anti-inflammatory	IL-1RA	7824.31 (4990.31)	1.85 (20.97)	**0.0002 (−3.76)**	334.44	18.10 (79.38)
	IL-4	0.59 (0.66)	0.05 (0.00)	**0.0001 (−3.84)**	10.37	0.108 (0.035)
	IL-10	3.37 (4.48)	0.08 (0.00)	**0.0001 (−3.88)**	46.69	0.33 (1.13)
	IL-13	35.26 (20.57)	4.53 (0.00)	**0.0001 (−3.88)**	8.71	4.44 (0.38)
Cartilage degradation	ACG	2077.5 (490.0)	560.0 (655.0)	0.06 (−1.83)	2.69	2775.0 (480.0)
	CTX-2	438,005 (171054)	100,805 (452484)	0.16 (−1.39)	1.38	385,367 (151414)
Metabolic	bFGF	18.68 (25.26)	26.62 (3.34)	0.53 (0.62)	0.51	24.78 (27.33)
	TGF-β1	2588.24 (4233.90)	2923.56 (4104.46)	0.79 (−0.26)	0.88	2710.86 (2266.59)
	TGF-β2	62.75 (45.25)	152.95 (1554.52)	0.30 (1.02)	0.10	22.04 (25.25)
	TGF-β3	3.22 (2.07)	6.35 (0.00)	**0.0025 (3.02)**	0.48	0.85 (0.41)

*Abbreviation: IQR: interquartile range*. Wilcoxon rank-sum test with z-value and P-value. Data in bold indicate a significant difference in cytokine levels. Several cytokines were significantly elevated in fractured knees compared to contralateral healthy knees

### Correlation of cytokine levels with clinical outcomes 3 and 12 months postoperative

Interestingly, the pain visual analog scale (VAS) at rest and VAS at mobilization after 12 months demonstrated numerous significant correlations with cytokine levels. VAS at rest after 12 months correlated with IL-6, MMP-3, TNF- α, and b-FGF. VAS at mobilization after 12 months correlated with IL-2, IL-8, TNF- α, IL-1RA, IL-10, TGF- β2, and IL-1β. Furthermore, the metabolic mediator cytokine TGF-β1 correlated with several clinical outcomes such as KOOS at 12 months, EQ. 5D at 12 months, mobilization level at 12 months and EQ. 5D VAS at 12 months (Table 4). The significant correlations are highlighted in Table 4.

**Table 4:** correlation of cytokine levels versus clinical outcomes 3 and 12 months after knee surgery

	Cytokines	KOOS 3 months *P*-value (coef.)	KOOS 12 months *P*-value (coef.)	EQ 5D 3 months *P*-value (coef.)	EQ 5D 12 months *P*-value (coef.)	VAS 3 months, rest *P*-value (coef.)	VAS 12 months, rest *P*-value (coef.)	VAS 3 months, mobilization *P*-value (coef.)	VAS 12 months, mobilization *P*-value (coef.)	Mobilization level 3 months *P*-value (coef.)	Mobilization level 12 months *P*-value (coef.)	Swelling 3 months *P*-value (coef.)	Swelling 12 months *P*-value (coef.)	EQ 5 D VAS 3 months *P*-value (coef.)	EQ 5 D VAS 12 months *P*-value (coef.)
Pro-inflammatory	IL-1α	–	–	–	0.03 (0.65)	–	–	–	–	–	–	–	–	–	0.04 (0.60)
	IL-1β	–	–	–	–	–	–	–	0.0015 (−0.55)	–	–	–	–	–	–
	IL-2	–	–	–	–	–	–	–	0.0019 (0.82)	–	–	–	–	–	–
	IL-6	–	–	–	–	–	0.04 (−0.62)	0.03 (0.69)	–	–	–	–	–	–	–
	IL-8	–	–	–	–	–	–	–	0.018 (0.72)	–	–	–	–	–	–
	IL-12p70	–	–	–	–	–	–	–	–	–	–	–	–	–	–
	TNF-α	–	–	–	–	–	0.04 (0.60)	–	0.02 (0.68)	–	–	–	–	–	–
	TNF-β		–	–	–	–	–	–	–	–	–	–	0.04 (−0.61)	–	–
	IFN-y	–	–	–	–	–	–	–	–	–	–	–	–	–	–
	MMP-1	–	–	–	–	–	–	–	–	–	–	–	–	–	–
	MMP-3	–	–	–	–	–	0.0053 (−0.77)	–	–	–	–	–	–	–	–
	MMP-9	–	–	–	–	–	–	–	–	–	–	–	–	–	–
Anti-inflammatory	IL-1RA	–	–	–	–	–	–	–	0.0094 (0.73)	–	–	–	–	–	–
	IL-4	–	–	–	–	–	–	–	–	–	–	–	–	–	–
	IL-10	–	–	–	–	–	–	–	0.047 (0.60)	–	–	–	–	–	–
	IL-13	–	0.036 (0.63)	–	–	–	–	–	–	–	–	–	–	–	–
Cartilage degradation	ACG	–	–	–	–	–	–	–	–	–	–	–	–	–	–
	CTX2	–	–	–	–	–	–	–	–	–	–	–	–	–	–
Metabolic	b-FGF	–	–	–	–	–	0.0043 (0.78)	–	–	–	–	–	–	–	–
	TGF-β1	–	0.0039 (0.78)	–	0.007 (0.75)	–	–	–	–	–	0.0051 (0.77)	–	–	–	0.02 (0.65)
	TGF-β2	–	–	–	–	–	–	–	0.047 (0.60)	–	–	–	–	–	–
	TGF-β3	–	–	–	–	–	–	–	–	–	–	0.0071 (0.85)	–	–	–

Abbreviation

Coef.: Coefficient value (range from −1 to 1 and indicates a one-unit change in the variables will result in an x unit change in the outcome scores) KOOS: Knee injury and Osteoarthritis Outcome Score VAS: visual analog scale

Spearman’s rank correlation test was performed, and only P-values below 0.05 were reported. “-“indicate no statistical significance

### Cytokine levels in the fractured knee were significantly correlated with cytokine levels in plasma

There was a significant positive correlation between pro-inflammatory cytokines TNF-α (*P* = 0.02, *ρ* = 0.77), TNF-β (*P* = 0.01, *ρ* = 0.76) and a negative correlation with IL-6 (*P* = 0.02, *ρ* = − 0.72) with their corresponding level in plasma. We also found a positive correlation between the metabolic mediator TGF-β2 (*P* = 0.01, *ρ* = 0.76) and a negative correlation of TGF-β3 (*P* = 0.03, *ρ* = − 0.69). No statistically significant correlation was observed for all anti-inflammatory cytokines and cartilage degradation products, as well as the remaining pro-inflammatory cytokines and metabolic mediators (Supplement 1).

### Correlation of fracture reduction with clinical outcomes

Supplements 2 and 3 show a significant correlation for EQ. 5D and EQ. 5D VAS after 12 months with fixation and PPTS difference. Not unexpected, very few clinical outcomes had a significant correlation with fracture reduction (Supplements 2,3). Additional information and description regarding injury mechanism and fracture classification are presented in Supplements 4 and 5.

## Discussion

This prospective cohort of 12 patients with acute intra-articular proximal tibia fractures is, to the best of our knowledge, the largest study to report on cytokine levels using the contralateral knee joint for comparison and the association of intra-articular cytokine levels with 3- and 12-month postoperative clinical outcomes. Acute inflammatory cytokines have been implicated as one of many factors contributing to joint degeneration in intra-articular joint injury leading to PTOA [1–3, 5, 6].

This study found a significant increase of nearly all the pro-inflammatory cytokines investigated, such as IL-1β, IL-2, IL-6, IL-8, IL-12p70, TNF-α, IFN-y, MMP-1, MMP-3, and MMP-9. Moreover, all anti-inflammatory cytokines IL-1RA, IL-4, IL-10, and IL-13 were elevated significantly compared to the healthy contralateral knee. Only the metabolic mediator cytokine TGF-β3 had a significant decrease in its level. No cartilage degradation products ACG and CTX-2 proved to be significantly elevated. Furthermore, several pro- and anti-inflammatory cytokines and metabolic mediators were correlated to 12-month postoperatively clinical outcomes. The results of this study supports the current understanding of the inflammatory response behind the development of PTOA and could supplement the surgical treatment of intra-articular fractures [2, 6, 16–19].

### Cytokines were significantly increased in the fractured knee compared to the contralateral knee

The production of IL-6 in joints usually occurs as a response to increased cytokine levels of IL-1β and TNF-α [19, 20]. Previous studies have confirmed the synergy of IL-1β and TNF-α to decrease the synthesis of the building blocks of the extracellular matrix (ECM) and increase the synthesis of proteolytic enzymes such as MMPs [19, 21]. Moreover, the pro-inflammatory cytokines have been shown to stimulate cells to synthesis other inflammatory cytokines, thus promoting their production in a self-propelling way [6, 16, 19, 20]. The cytokines observed to act this way are IL-1β, IL-6, IL-8, and TNF-α [16, 19]. In this study, IL-6 had the largest fracture/contralateral knee ratio (2618) in our data (Table 3). IL-6 has been shown to promote osteoclast differentiation and bone resorption [6, 19]. Haller et al. demonstrated a significantly elevated level of IL-6 in the injured knee, which persisted through to the second time point of aspiration [6]. A large upregulation of IL-6 might have an impact over time and, therefore, play an important role in the development of PTOA. We also found that IL-8, IL-1β, TNF-α, and MMP-1 had a higher ratio than the other cytokines measured (212, 40, 20, and 74) compared to levels in the healthy knee (Table 3). Lieberthal et al. speculate that the lack of adequate, post-injury control of the pro-inflammatory cytokines in some patients maintains the chronic inflammation and tissue damage that leads to PTOA [18]. The possible synergy and autocrine behavior of these cytokines might have an important role in the development of the acute phase of chronic inflammation post-injury due to their large upregulation [19].

Another important finding was that the anti-inflammatory cytokines IL-1RA and IL-10 had a more than 40-fold increase (334 and 46) in their ratio (Table 3). IL-10 has been shown to stimulate the production of IL-1RA, type 2 collagen and ACG production as well as inhibiting MMPs, chondrocyte apoptosis and downregulating IL-1β and TNF-α [19, 22]. The presence of increased anti-inflammatory cytokines might suggest that a chondroprotective milieu is stimulated as a response to the pro-inflammatory cytokines. Furthermore, the lack of increased concentration of the metabolic mediators, indicate that the cartilage was not influenced by the inflammation, at the time of aspiration (Table 3). However, further research would be needed to investigate the balance between pro- and anti-inflammatory cytokines in the acute milieu in intra-articular knee fractures to understand the timeline and mechanism where the inflammatory process tends towards a chronic state of inflammation leading to PTOA.

### Clinical outcomes after 12 months correlate with several cytokines

Interestingly, we found that several pro-inflammatory cytokines were correlated with 12-month postoperative clinical outcomes, indicating that upregulation of acute pro-inflammatory cytokines might correlate with knee pain and the overall quality of life (Table 4). This, in contrast to the fact that all clinical parameters recorded in this study were improved between the 3- and 12-month follow-up (Table 2). Surprisingly, anti-inflammatory cytokines IL-1RA, IL-10, and IL-13 were positively correlated with 12-month postoperative outcomes (Table 4). These findings suggest that a response to the pro-inflammatory cytokines might have an impact on the clinical outcome as well. A similar study conducted on tibial plateau fractures by Haller et al. found that the anti-inflammatory cytokines IL-1RA and IL-10 remained elevated at a second time point of aspiration, and that the concentration of IL-1RA increased [6].

The clinical outcome VAS in activity at 12 months, correlated with numerous cytokines, such as IL-1β, IL-2, IL-8, TNF-α, IL-1RA, IL-10, and TGF-β1 (Table 4). All the cytokines except TGF-β1 were significantly elevated in the fractured knee (Table 3). Surprisingly, the levels of cytokines IL-1RA and IL-10 were positively correlated with VAS in activity at 12 months. This finding was unexpected and suggests that IL-1RA and IL-10 may have a role in developing early symptoms of PTOA. Further research would be needed to identify the potential negative role of anti-inflammatory cytokines in the joint milieu, before drawing conclusions. These findings and the findings of Haller et al. raise the question of the role of transient versus prolonged cytokine elevation, whether it is pro- or anti-inflammatory and the role of these cytokines on the chondrocyte physiology [6]. Lastly, in this study, the cytokine level of cartilage degradation products ACG and CTX-2 had no significant correlation with outcomes and was slightly but not significantly elevated in the fractured knee (Tables 3,4). This result may be explained be the early timing of aspiration. Another possible explanation for this is that the cartilage is slightly resistant against the initial inflammation caused by the fracture. This only becomes a problem when the inflammation persists, and the concentration of cartilage degradation products increase. Further research should investigate the possible correlation of inflammatory cytokines with clinical outcomes in a larger population and serial sampling during follow-ups to provide more information about the joint milieu in intra-articular knee fractures.

### Monitoring cartilage health with serum levels of cytokines

We found that an elevation of IL-6 was negatively correlated to plasma IL-6 level (Supplement 1). This finding suggests that an upregulation of IL-6 may be detected in the fractured joint due to the timing of sampling collection. Measurement of the levels of IL-6 in serum at a second time point could show an increase in its level as it has been shown to have a persistently elevated level in the SF collected from fractured knees [6]. However, the concentration of cytokines in serum is not only dependent of the inflammatory process in the fractured knee, but the overall regulation of cytokines in the body. In this study, TNF-α and TNF-β where both positively correlated with serum levels, while TNF-α was significantly elevated in the fractured knee (Table 3, Supplement 1). Furthermore, TNF-α has been shown to stimulate the production of other cytokines [19]. The positive correlation with VAS at 12 months in this study could open the possibility of investigating it as a potential biomarker of early-onset PTOA during follow-up (Table 4).

Lastly, we found that the levels of the metabolic mediators TGF-β2 and -β3 were correlated with SF of the fractured knee, with only TGF-β3 showing a significant decrease in the injured knee (Table 3, Supplement 1). In vitro studies have shown that the TGF-β signaling pathway switches its protective role in normal cartilage to a more damaging role in advanced OA. Both overactivity and lack of activity could suggest that only a narrow range of TGF-β can maintain cartilage health [23, 24]. Therefore, TGF-β mediators could perhaps be used to monitor the level of cartilage health through serum sampling. However, it might take some time before an elevation of cytokines in the fractured knee joint causes an increase (or decrease) of serum cytokines. Therefore, further investigation where sampling serum is a part of the follow-up, could reveal suitable cytokines that could serve as a diagnostic biomarker of early-onset PTOA.

A discussion of the last exploratory outcome would go beyond the scope of this paper. However, it is worth mentioning that future studies should, if possible, include the measurement of these variables presented in this paper during follow-up, as they may perhaps influence or help predict clinical outcomes.

### Limitations

The first limitation of this study is the small sample size (*n* = 12). It is possible that with a larger study, these cytokines might contribute additional information regarding clinical outcomes. However, we have demonstrated that some cytokines correlate with clinical outcome after 12 months. Second, the follow-up period might not be long enough to see any significant changes in outcome. Therefore, future studies with follow-up beyond 12 months are suggested. Third, we can’t be sure that we didn’t alter the concentration of proteins in some knees more than others, since the volume of SF in each knee before injecting the saline was unknown. Fourth, due to the relatively small number of patients included, a multiple comparison cannot statistically be justified. However, our results may generate additional hypotheses for future research. Lastly, 1 patient was lost to follow-up after 12-months.

## Conclusion

In conclusion, we found an elevation of nearly all pro-inflammatory cytokines investigated, including IL-1β, IL-2, IL-6, IL-8, IL-12p70, TNF-α, IFN-y, MMP-1, MMP-3, and MMP-9 and a simultaneous elevation of anti-inflammatory cytokines, such as IL-1RA, IL-4, IL-10, and IL-13, following intra-articular proximal knee fractures compared to healthy contralateral joints. The results of this study indicate that there is an initiation of an inflammatory process after intra-articular knee fractures, which is characterized by the elevation of both pro- and anti-inflammatory cytokines. No difference was found for IL-1α, TNF-β, cartilage degradation proteins ACG and CTX-2, and metabolic mediators bFGF, TGF-β1 and -β2. There was no sign of cartilage damage within the timeframe from injury to operation. Furthermore, several pro- and anti-inflammatory cytokines and metabolic mediators were correlated to 12-month postoperative clinical outcomes, especially in regards of pain perception. Lastly, TNF-α and TGF-β mediators could serve as a diagnostic biomarker during follow-up.

### Clinical relevance

This prospective cohort contributes information regarding the initial inflammatory cascade after an acute intra-articular knee fracture and the possible correlation of cytokines with clinical outcomes. It supports the current understanding of the inflammatory mechanism behind the development of PTOA and will supply information towards a potential supplement to the current surgical treatment of intra-articular fractures.

## Supplementary Information

**Additional file 1.**

## Data Availability

All data are hosted at OPEN (Odense Patient Explorative Network), which allows data sharing upon request. The dataset used in this study is available upon request from the corresponding author on reasonable request. https://www.sdu.dk/da/om_sdu/institutter_centre/klinisk_institut/forskning/forskningsenheder/open.Aspx

## Abbreviations

ACG: Aggrecan
ASA: American Society of Anesthesiologists
AO: Arbeitsgemeinschaft für Osteosynthesefragen
BMI: Body mass index
bFGF: Basic fibroblast growth factor
CTX-2: C-terminal telopeptides of type 2 collagen
EDTA: Ethylenediaminetetraacetic acid
ELISA: Enzyme-linked immunosorbent assay
EQ 5D: Euroqol 5D questionnaire
HS: Hagen Schmal.
IFN-y: Interferon gamma.
IJI: Imran Jamal Iversen.
IL: Interleukin.
IL-1RA: Interleukin 1 receptor antagonist.
IQR: Interquartile range.
ISS: Injury Severity Score.
ITPL: Intact tibial plateau line.
JSN: Joint space narrowing.
KLS: Kellgren-Lawrence scale.
KOOS: Knee injury and Osteoarthritis Outcome Score.
LATS: Long axis of tibial shaft.
LAT: Longitudinal axis of the tibia.
MLSO: Medial/lateral step off.
MMP: Matrix metalloproteinase.
MPTA: Medial proximal tibial angle.
OA: Osteoarthritis.
PTOA: Posttraumatic osteoarthritis.
PPTS: Proximal posterior tibial slope.
PWTP: Putative widening of the tibial plateau.
qq-plot: Quantile-quantile (q-q)-plot.
RPM: revolutions per minute.
SF: Synovial fluid.
TGF: Transforming growth factor.
TMP: That Minh Pham.
TNF: Tumor necrosis factor.
VAS: visual analog scale.

## Declarations

This study was conducted in accordance with the principles of the Declaration of Helsinki and approved by the Regional Committees on Health Research Ethics for Southern Denmark. Patients was informed of the project both in writing and orally prior to signing the informed consent for participation in the project. Written, informed consent was obtained from all patients.
