# Supplementary material for: Do acute inflammatory cytokines affect 3- and 12-month postoperative functional outcomes–a prospective cohort study of 12 patients with proximal tibia fractures
Source: BMC Musculoskelet Disord. 2021 Apr 10;22:342. doi: 10.1186/s12891-021-04207-7 (PMC8035750; doi:10.1186/s12891-021-04207-7)
Supplement: Supplementary file 1 — Additional file 1. [file 12891_2021_4207_MOESM1_ESM.docx]

| Supplement 1: Correlation of cytokine levels in fractured knees and plasma | | | | | | |
| --- | --- | --- | --- | --- | --- | --- |
|  | **Cytokine**  **levels** | **Fractured knees (pg/mL)**  **Median (IQR)** | **Serum (pg/ml)**  **Median (IQR)** | **P-value (rho)**  **(fractured vs serum)** |  |  |
| Pro-inflammatory | IL-1α | 0.01345 (0.01226) | 0.00942 (0.02051) | 0.89 (–0.05) |  |  |
|  | IL-1β | 5.16 (2.86) | 0.665 (0.465) | 0.23 (0.44) |  |  |
|  | IL-2 | 3.43 (2.21) | 0.710 (0.005) | 0.69 (–0.15) |  |  |
|  | IL-6 | 1630.62 (865.54) | 5.93 (3.17) | **0.02 (–0.72)** |  |  |
|  | IL-8 | 379.21 (309.79) | 3.35 (4.41) | 0.91 (0.04) |  |  |
|  | IL-12p70 | 6.95 (5.16) | 0.71 (0.06) | 0.25 (–0.42) |  |  |
|  | TNF-α | 8.92 (7.77) | 2.61 (1.42) | **0.02 (0.77)** |  |  |
|  | TNF-β | 0.0000419 (0.000116) | 0.0000426 (7.00e-07) | **0.01 (0.76)** |  |  |
|  | IFN-y | 28.81 (24.51) | 10.65 (12.07) | 0.30 (0.38) |  |  |
|  | MMP-1 | 426290.2 (804764.8) | 1649.53 | 0.26 (0.41) |  |  |
|  | MMP-3 | 342739.1 (675480.8) | 6812.93 (1817.63) | 0.63 (0.18) |  |  |
|  | MMP-9 | 76405.31 (56070.87) | 49956.95 (79704.52) | 0.79 (0.10) |  |  |
| Anti-inflammatory | IL-1RA | 7824.31 (4990.31) | 18.10 (79.38) | 0.57 (0.21) |  |  |
|  | IL-4 | 0.59 (0.66) | 0.108 (0.035) | 0.96 (–0.01) |  |  |
|  | IL-10 | 3.37 (4.48) | 0.33 (1.13) | 0.75 (0.12) |  |  |
|  | IL-13 | 35.26 (20.57) | 4.44 (0.38) | 0.69 (0.15) |  |  |
| Cartilage degradation | ACG | 2077.5 (490.0) | 2775.0 (480.0) | 0.54 (0.23) |  |  |
|  | CTX-2 | 438005 (171054) | 385367 (151414) | 0.35 (0.35) |  |  |
| Metabolic | bFGF | 18.68 (25.26) | 24.78 (27.33) | 0.40 (0.31) |  |  |
|  | TGF-β1 | 2588.24 (4233.90) | 2710.86 (2266.59) | 0.51 (0.25) |  |  |
|  | TGF-β2 | 62.75 (45.25) | 22.04 (25.25) | **0.01 (0.76)** |  |  |
|  | TGF-β3 | 3.22 (2.07) | 0.85 (0.41) | **0.03 (–0.69)** |  |  |
| Abbreviation: IQR: interquartile range. Spearman´s rank correlation with P-value and rho. | | | | | | |

| Supplement 2: correlation of fracture reduction versus clinical outcomes 3 and 12 months after knee surgery. | | | | | | |  |
| --- | --- | --- | --- | --- | --- | --- | --- |
|  |  | **KOOS 3 months**  P-value (coef.) | **KOOS 12 months**  P-value (coef.) | **EQ5D 3 months**  P-value (coef.) | **EQ5D 12 months**  P-value (coef.) | **VAS 3 months, rest**  P-value (coef.) | **VAS 12 months, rest**  P-value (coef.) |
|  | Schatzker | - | - | - | - | - | - |
|  | Fixation | - | - | - | 0.03 (0.69) | - | - |
|  | AO score | - | - | - | - | - | - |
|  | MPTA dif. | - | - | - | - | - | - |
|  | PPTS dif. | - | - | - | 0.03 (–0.74) | - | - |
|  | JSNM dif. | - | - | - | - | - | - |
|  | JSNL dif. | - | - | - | - | - | - |
|  | K-L score | - | - | - | - | - | - |
|  | PWTP dif. | - | - | - | - | - | - |
|  | MLSO dif. | 0.03 (0.75) | - | - | - | - | - |
|  | GAP dif. | - | - | - | - | - | - |

| Abbreviation:  Coef.: Coefficient value (range from –1 to 1 and indicate one-unit change in the variables will result in x units change in the outcome scores),  KOOS: Knee injury and Osteoarthritis Outcome Score, VAS: visual analogue scale, AO score: Arbeitsgemeinschaft für Osteosynthesefragen, dif.: difference between 3 and 12 months, MPTA: Medial Proximal Tibial Angle, PPTS: Proximal Posterior Tibial Slope, JSNM: Joint Space Narrowing Medial, JSNL: Joint Space Narrowing Lateral, K-L score: Kellgren-Lawrence score, PWTP: Putative Widening of the Tibial Plateau, MLSO: Medial/Lateral Step-Off.  Spearman´s rank correlation test was performed and only P-values below 0.05 were reported. “-“indicate no statistical significance. |
| --- |

| Supplement 3: correlation of fracture reduction versus clinical outcomes 3 and 12 months after knee surgery | | | | | | |  |  |  |
| --- | --- | --- | --- | --- | --- | --- | --- | --- | --- |
|  |  | **VAS 3 months, mobilization**  P-value (coef.) | **VAS 12 months, mobilization**  P-value (coef.) | **Mobilization level 3 months**  P-value (coef.) | **Mobilization level 12 months**  P-value (coef.) | **Swelling 3 months**  P-value (coef.) | **Swelling 12 months**  P-value (coef.) | **EQ5D VAS 3 months**  P-value (coef.) | **EQ5D VAS 12 months**  P-value (coef.) |
|  | Schatzker | - | - | - | - | - | - | - | - |
|  | Fixation | - | - | - | - | - | - | - | 0.04 (0.67) |
|  | AO score | - | - | - | - | - | - | - | - |
|  | MPTA dif. | 0.02 (–0.76) | - | - | - | - | - | - | - |
|  | PPTS dif. | - | - | - | - | - | - | - | 0.01 (0.80) |
|  | JSNM dif. | - | - | - | - | - | - | - | - |
|  | JSNL dif. | - | - | - | - | - | - | - | - |
|  | K-L score | - | - | - | - | - | - | - | - |
|  | PWTP dif. | - | - | - | - | - | - | 0.03 (–0.74) | - |
|  | MLSO dif. | - | - | - | - | - | - | - | - |
|  | GAP dif. | - | - | - | - | - | - | - | - |

| Abbreviation:  Coef.: Coefficient value (range from –1 to 1 and indicate one-unit change in the variables will result in x units change in the outcome scores),  VAS: visual analogue scale, AO score: Arbeitsgemeinschaft für Osteosynthesefragen, dif.: difference between 3 and 12 months, MPTA: Medial Proximal Tibial Angle, PPTS: Proximal Posterior Tibial Slope, JSNM: Joint Space Narrowing Medial, JSNL: Joint Space Narrowing Lateral, K-L score: Kellgren-Lawrence score, PWTP: Putative Widening of the Tibial Plateau, MLSO: Medial/Lateral Step-Off.  Spearman´s rank correlation test was performed and only P-values below 0.05 were reported. “-“indicate no statistical significance. |
| --- |

| Supplement 4: fracture classification with additional surgeries | | | | | | | | | | | |
| --- | --- | --- | --- | --- | --- | --- | --- | --- | --- | --- | --- |
|  | **Patient** | **AO classification** | | |  | **Additional surgery at 12-month** | | | |  | |
| 1 | | | 41B3.1 |  | | | Removal of osteosynthesis material | | |  |  |
| 2 | | | 41B3.1 |  | | | None | | |  |  |
| 3 | | | 41C2.1 |  | | | Arthroscopic partial synovectomy, removal of cannulated screws, partial skin transplantation of knee, TKA | | |  |  |
| 4 | | | 41C3.1 |  | | | Arthroscopic partial synovectomy | | |  |  |
| 5 | | | 41B3.2 |  | | | None | | |  |  |
| 6 | | | 41B3.1 |  | | | Cosmetical abdominoplasty | | |  |  |
| 7 | | | 41C2.1 |  | | | None | | |  |  |
| 8 | | | 41B2.1 |  | | | Removal of osteosynthesis material | | |  |  |
| 9 | | | 41B1.1 |  | | | None | | |  |  |
| 10 | | | 41C2.2 |  | | | None | | |  |  |
| 11 | | | 41C2.1 |  | | | None | | |  |  |
| 12 | | | 41B3.3 |  | | | None | | |  |  |
|  | | |  | | | |  |  |  | |  |
| Abbreviation: AO: Arbeitsgemeinschaft für Osteosynthesefragen, TKA: total knee alloplastic | | | | | | | | | | | |

| Supplement 5: Injury mechanism and comorbidities | | | | | | | | | | | | | |
| --- | --- | --- | --- | --- | --- | --- | --- | --- | --- | --- | --- | --- | --- |
|  | **Patient** | **AO classification** | | | **Velocity of injury** | **Description of trauma mechanism** | | | **Comorbidities ACL/PCL lesion** | | | | |
| 1 | | | 41B3.1 | High velocity | | | Fell while cycling | | | Diabetes Mellitus, HT, HC | | None | |
| 2 | | | 41B3.1 | Low velocity | | | Fell from staircase below 1 meter | | | None | | None | |
| 3 | | | 41C2.1 | High velocity | | | Fell driving scooter | | | HT, arrythmia | | None | |
| 4 | | | 41C3.1 | Low velocity | | | Fell from garden chair | | | Alcoholic | | None | |
| 5 | | | 41B3.2 | Low velocity | | | Fell from playground swing | | | None | | None | |
| 6 | | | 41B3.1 | Low velocity | | | Fell from staircase above 1 meter | | | HT | | None | |
| 7 | | | 41C2.1 | Low velocity | | | Fell playing football with rotation of knee | | | None | | None | |
| 8 | | | 41B2.1 | Low velocity | | | Fell in shower | | | NSTEMI, HT | | None | |
| 9 | | | 41B1.1 | High velocity | | | Fell driving scooter | | | None | | None | |
| 10 | | | 41C2.2 | Low velocity | | | Fell down staircase below 1 meter | | | None | | None | |
| 11 | | | 41C2.1 | High velocity | | | Drove motorcycle into car at 60 km/h | | | Diabetes Mellitus | | None | |
| 12 | | | 41B3.3 | Low velocity | | | Fell | | | None | | None | |
|  | | |  | | | |  |  | | |  | |  |
| Abbreviation: AO: Arbeitsgemeinschaft für Osteosynthesefragen, km/h: kilometers per hour, HT: Hypertension, NSTEMI: Non-ST-elevation myocardial infarction, HC: Hypercholesterolemia, ACL: Anterior Cruciate Ligament, PCL: Posterior Cruciate Ligament | | | | | | | | | | | | | |
